# Supplementary material for: A Comprehensive Assessment of Ultraviolet-Radiation-Induced Mutations in Flammulina filiformis Using Whole-Genome Resequencing
Source: J Fungi (Basel). 2024 Mar 20;10(3):228. doi: 10.3390/jof10030228 (PMC10971301; doi:10.3390/jof10030228)
Supplement: Supplementary file 1 [file jof-10-00228-s001.zip › Supplementary Material S8/KEGG annotation/out/64381550635650.os/KO/out_map/map05143.html]

KEGG PATHWAY: African trypanosomiasis - Reference pathway


|  |  |
| --- | --- |
| **African trypanosomiasis - Reference pathway** |  |

[
Pathway menu
| Organism menu
| Pathway entry
| Show description
| User data mapping
]

Trypanosoma brucei, the parasite responsible for African trypanosomiasis (sleeping sickness), are spread by the tsetse fly in sub-Saharan Africa. The parasites are able to pass through the blood-brain barrier and cause neurological damage by inducing cytokines like TNF alpha, IFN gamma, and IL1. These cytokines and other metabolites such as nitric oxide and somnogenic prostaglandin D2 disturb circadian rhythms in patients with African trypanosomiasis.

|  |  |
| --- | --- |
| Reference pathway | 100% |
